# A role for artificial intelligence in molecular imaging of infection and inflammation

**DOI:** 10.1186/s41824-022-00138-1

**Published:** 2022-09-01

**Authors:** Johannes Schwenck, Manfred Kneilling, Niels P. Riksen, Christian la Fougère, Douwe J. Mulder, Riemer J. H. A. Slart, Erik H. J. G. Aarntzen

**Affiliations:** 1 Department of Nuclear Medicine and Clinical Molecular Imaging, Eberhard Karls University, Tübingen, Germany; 2 Department of Preclinical Imaging and Radiopharmacy, Werner Siemens Imaging Center, Eberhard Karls University, Röntgenweg 13, 72076 Tübingen, Germany; 3 Cluster of Excellence iFIT (EXC 2180) “Image-Guided and Functionally Instructed Tumor Therapies”, Eberhard Karls University, Tübingen, Germany; 4 Department of Dermatology, Eberhard Karls University, Tübingen, Germany; 5 Department of Internal Medicine, Radboud University Medical Center, Nijmegen, The Netherlands; 6 Department of Nuclear Medicine and Molecular Imaging, University Medical Center Groningen, Groningen, The Netherlands; 7 Department of Biomedical Photonic Imaging, Faculty of Science and Technology, University of Twente, Enschede, The Netherlands; 8 Department of Medical Imaging, Radboud University Medical Center, Nijmegen, The Netherlands; 9 Department of Internal Medicine, University of Groningen, University Medical Center Groningen, Groningen, The Netherlands

## Abstract

The detection of occult infections and low-grade inflammation in clinical practice remains challenging and much depending on readers’ expertise. Although molecular imaging, like [^18^F]FDG PET or radiolabeled leukocyte scintigraphy, offers quantitative and reproducible whole body data on inflammatory responses its interpretation is limited to visual analysis. This often leads to delayed diagnosis and treatment, as well as untapped areas of potential application. Artificial intelligence (AI) offers innovative approaches to mine the wealth of imaging data and has led to disruptive breakthroughs in other medical domains already. Here, we discuss how AI-based tools can improve the detection sensitivity of molecular imaging in infection and inflammation but also how AI might push the data analysis beyond current application toward predicting outcome and long-term risk assessment.

## Introduction

Artificial intelligence (AI) is considered to be the key to precision medicine and transforming health care (Denny and Collins 2021). In line with other imaging disciplines, such as microscopy (Meijering et al. 2016) and pathology (Colling et al. 2019; Laak et al. 2021), images obtained from routine clinical procedures represent rich and minable datasets on specific tissue characteristics (Gillies et al. 2016; Aerts et al. 2014). This realization urged the development of AI-based technologies to exploit these wealthy data sources (Parmar et al. 2018; Hosny et al. 2018). Although practical issues concerning data sharing, data safety and standardization are yet to be resolved (He et al. 2019; Currie and Hawk 2021), ongoing developments in AI will drive its implementation in the field of medical imaging (Currie and Rohren 2021). When it comes to application of AI-based technology for nuclear imaging modalities such as positron emission tomography (PET) and single-photon emission tomography (SPECT), excellent reviews which discuss modality-specific potential and limitations are available from the recent literature (Hatt et al. 2021; Uribe et al. 2019; Zukotynski et al. 2021; Decuyper et al. 2021). A key asset of nuclear imaging modalities is their whole body field-of-view and hence the capacity to quantify the distribution of tracers targeting specific biological processes where several organs and tissues are involved. Furthermore, dynamic imaging in nuclear medicine offers the possibility to temporally resolve systemic processes. Both aspects of nuclear imaging are extremely useful to develop a ‘systems biology’ approach based on nuclear imaging to characterize host’ immune responses in infection and inflammation.

AI methodology is evolving rapidly and it is beyond the scope of this review to provide a comprehensive overview on current concepts in image analysis. In general, AI-based approaches can be divided into supervised and unsupervised learning methods. Supervised learning requires data which is considered a ground truth or a gold standard, like histopathology. Supervised learning therefore is a mathematical way to approximate a model using a labeled training dataset which is then optimized in iterative steps. Typically, validation and test datasets are needed to assess the accuracy of the developed model. Unsupervised learning is trained to recognize patterns in unlabeled data without ground truth information. In unsupervised learning, algorithms are searching for regularities that can be used to define relationships like groups with similar features in an unlabeled dataset. Furthermore, unsupervised learning methods are used for capturing noise in data or to generate new data samples. Clustering methods like k-means are common unsupervised approaches to find patterns between data points in a dataset. More sophisticated approaches use, for example, trained neural networks which allow to model more complex relationships with only little assumptions (LeCun et al. 2015).

While in its early days, now is the time to also consider the potential roles of AI specifically in molecular imaging of infection and inflammation. ‘Precision medicine’ in the field of inflammation translates to early identification of patients at risk for inflammatory diseases and tailored treatment duration based on individual characteristics of a patients’ immune system.

In recent years it became evident that the activation of the immune system requires metabolic reprogramming, especially in regard to glucose metabolism (Gaber et al. 2017), thus in principle leads to effects measurable with 2-[^18^F]fluoro-2-deoxy-D-glucose ([^18^F]FDG) PET. Likewise, the spatial distribution of immune cells throughout the body determines the effectiveness of immune responses, which is assessable, for example, by radiolabeled leukocyte scintigraphy. While extensively studied in the aspect of cancer immunotherapy, these effects are similarly important in inflammatory diseases including infections, autoimmune disorders and atherosclerosis.

An important aspect of inflammation is the emerging concept of trained immunity: long-term functional reprogramming of the innate immune cells which co-determines responses to subsequent triggers (Netea et al. 2020a; Schultze et al. 2018). The development of trained immunity is determined by epigenetic reprogramming and profound rewiring of metabolic circuits in immune cells.

Current routine analysis of imaging techniques like [^18^F]FDG PET or radiolabeled leukocyte scintigraphy rely on the visual detection of foci in symptomatic patients, which heavily depends on the readers’ reference. Given the pivotal role of metabolic reprogramming in a range of inflammatory conditions, it emerges that at present only the tip of the iceberg of the available information is extracted out of the acquired data.

The recent advances in AI might push the analysis of current imaging techniques toward a more comprehensive understanding of inflammatory diseases including atherosclerosis and infections, to the detection of pathological immune responses even in asymptomatic patients on the long run.

In this short communication we propose three potential tasks for AI, ranging from practical to more hypothetical, where AI-based technologies can be applied to improve current practice.

### Artificial intelligence to improve detection

**Fig. 1:**
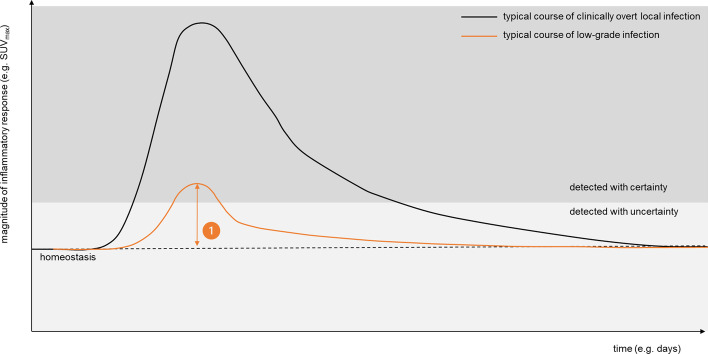
Artificial intelligence to improve detection. A graphical illustration of the typical dynamics of an immune response upon a trigger, with rapid increase, associated with increased glycolysis in effector cells which can be measured by [^18^F]FDG PET and expressed as maximum or mean standardized uptake values (SUVmax/mean). As soon as the causative trigger is cleared, inflammatory responses also include repair processes to gradually return to a state of tissue homeostasis

Over the past years, [^18^F]FDG PET/CT has established its central role in the diagnosis and follow-up of infectious diseases and inflammatory conditions (Slart et al. 2018a; Signore et al. 2019; Chakfe et al. 2020; Jamar et al. 2013). Based on the high sensitivity and favorable whole-body view, the range of clinical indications continues to expand and the questions to be addressed are increasingly complex. At these far-end applications in inflammation imaging, current PET technologies have probably met their limits of detection and discriminative power (Fig. 1).

This holds true particularly for infections at occult sites, low-grade infections or low-grade inflammatory conditions that are diagnosed late and treated with delay (Hipfl et al. 2021; Laohapensang et al. 2017; Talha et al. 2020). Although this concerns a minority of patients, these cases consume a lot of health care related services, multiple diagnostic tests are being performed and prolonged treatment is required with increased likelihood to encounter complications. For example, the diagnosis of infectious (native or prosthetic valve) endocarditis currently requires a composite of clinical, microbiological and imaging (ultrasound and [^18^F]FDG PET) to accomplish reasonable sensitivity and specificity (Chakfe et al. 2020; Habib et al. 2015). However, this investment in diagnostic accuracy is mandatory as we have learned that insufficient treatment of even these small intravascular infectious foci is associated with increased mortality (Jaltotage et al. 2021; Chirillo 2021).

Moreover, in the premise of ‘precision medicine,’ (intravenous) antibiotic treatment durations tend to be shortened (Berrevoets et al. 2019; Kouijzer et al. 2021) to avoid overtreatment and reduce health care expenditure. Thus, discriminating persistent foci of active infection from tissue remodeling at the end of antibiotic or anti-inflammatory treatment will be an increasingly relevant, but challenging task for [^18^F]FDG PET imaging.

As there is a need for improved detection of low-grade or localized infections, which inherent features of nuclear imaging techniques are limiting? In comparison with computed tomography (CT) nuclear imaging techniques suffer from long acquisition times, inherently resulting in motion artifacts. Furthermore, nuclear imaging is constraint by radiation dose and the associated safety considerations, which together with the urge to detect low-grade of localized infection, call for further optimization of detection efficiency of PET and SPECT systems. The introduction of new PET scanners with digital detector technology and a long-axial field of view of 100 cm or more provide significant improvements in this regard. These developments have the potential to significantly reduce the data acquisition times in PET, which makes a high-resolution whole-body PET scan in less than 5 min possible (Alberts et al. 2021; Filippi and Schillaci 2022). Besides that, the substantial advances of this generation of scanners also allow a better temporal and spatial resolution as well as reduction of the administered radiation dose.

The relatively poor spatial resolution of PET (3–4 mm) and SPECT (8–9 mm) hampers the accurate assessment of anatomical regions with respiratory and cardiac motion. This is particularly relevant for imaging subtle changes in signal intensity in the myocardium, for example when endocarditis is suspected; or discrimination of [^18^F]FDG uptake in aortic root complications after recent vascular graft surgery.

**Fig. 2:**
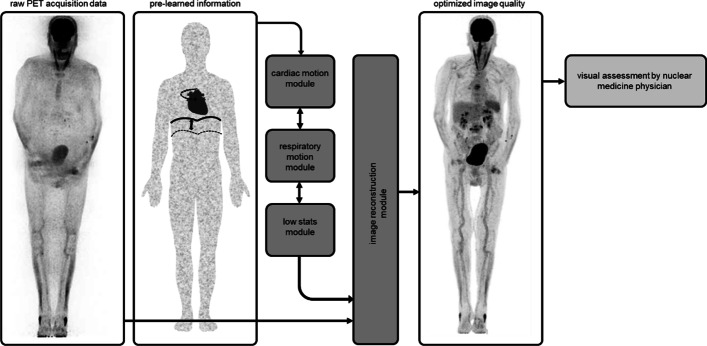
Potential AI workflow to improve image quality by cardiac and respiratory motion correction. The pre-learned, highly standardized movements of the heart and the lung can be integrated in the image reconstruction in order to optimize the image quality leading to advantages in the visual assessment by the nuclear medicine physician

Cardiac and respiratory motion, however, are highly standardized movements that can be modelled using AI-based technologies (Fig. 2). These are particularly suited to reconstruct images by incorporating previously learned information that compensate for motion. Indeed, data-driven approaches for PET image reconstruction that compensates for respiratory motion are increasingly available (Buther et al. 2016; Feng et al. 2019; Schleyer et al. 2011), paralleling developments in CT (Saeedan et al. 2021). Previously, electrocardiogram (ECG)-gated motion correction of [^18^F]-NaF uptake in coronary arteries in patients with myocardial infarction or stable angina had significant impact on lesion quantification (Rubeaux et al. 2016). Along the same line for endocarditis, more accurate detection of small infectious foci in the plane of cardiac valves would increase detection rates and allow better co-localization with findings on ultrasound or cardiac CT, which improves diagnostic accuracy (Hove et al. 2021). On a more general note, deep-learning methods to reconstruct whole body PET images without the signal-derived input for attenuation and scatter correction by its CT component are reported (Yang et al. 2021; Haggstrom et al. 2019), with non-inferior image quality, but much faster reconstruction times. Although large-scale comparative studies supporting AI-based motion correction are lacking, these approaches demonstrated that pre-learned information can be incorporated in AI-based reconstructions of PET acquisitions.

In addition to factors that affect the measured signal intensity, the presence of noise in PET and SPECT data impairs accurate visual assessment and diagnostic accuracy of scan images in low-count statistics and the detection of small foci with little signal-to-noise ratio (Minarik et al. 2020). For example, radiolabeled autologous leukocytes for SPECT imaging have long been used to detect infectious foci and with sufficient specificity to discriminate infection from inflammation (de Vries et al. 2010; Roca et al. 2010). However, its inferior image quality due to low-count statistics and high levels of noise resulted in a rapid take-over by [^18^F]FDG PET/CT for these indications (Jamar et al. 2013), as image quality and system sensitivity were preferred despite the use of a less specific tracer. AI-based technologies can be exploited to reduce noise in such settings, which will positively impact image interpretation. For PET imaging the assignment of a line-of-response (LOR) for accurate image reconstruction can be corrupted by non-perpendicular coincidences, resulting in uncertainties in positioning the input signal. A deep learning estimator has been developed to predict the depth-of-interaction of incoming photons in pixelated detectors, which resulted in improved performance (Zatcepin et al. 2020). Improved positioning of input signals for monolithic detectors has been improved using convolutional neural networks that integrates the charge of silicon photomultipliers to predict locations of non-perpendicular coincidences (He et al. 2021). Compton scattering in the detection crystal results in incorrectly assigned LORs and contributes to system noise for PET and SPECT imaging. Deep learning algorithms trained on Monte Carlo simulation data showed improved LOR recovery rates and sensitivity by including accurate position of events in image reconstruction (Bergeron et al. 2014). Furthermore, in PET imaging, prediction of adverse cardiovascular events has recently been studied through the implementation of transfer learning, which allows for data economization while boosting image recognition capabilities and broadening the horizon of network architectures that can be constructed (Vos et al. 2019).

Image denoising based on deep learning methods is applied in general image restoration in cases of low or lack of spatial input. Several studies have now shown potential to convert low-count to high-count PET or SPECT images using U-Net (Kaplan and Zhu 2019; Dietze et al. 2019) or ResNet (Gong et al. 2019; Cui et al. 2019) algorithms. Lastly, on the hardware site of development, the new generation of long-axis field-of-view PET scanners have an even better sensitivity (Alberts et al. 2021; Badawi et al. 2019) which can also be exploited to increase signal-to-noise ratio.

### Artificial intelligence to predict outcomes

**Fig. 3:**
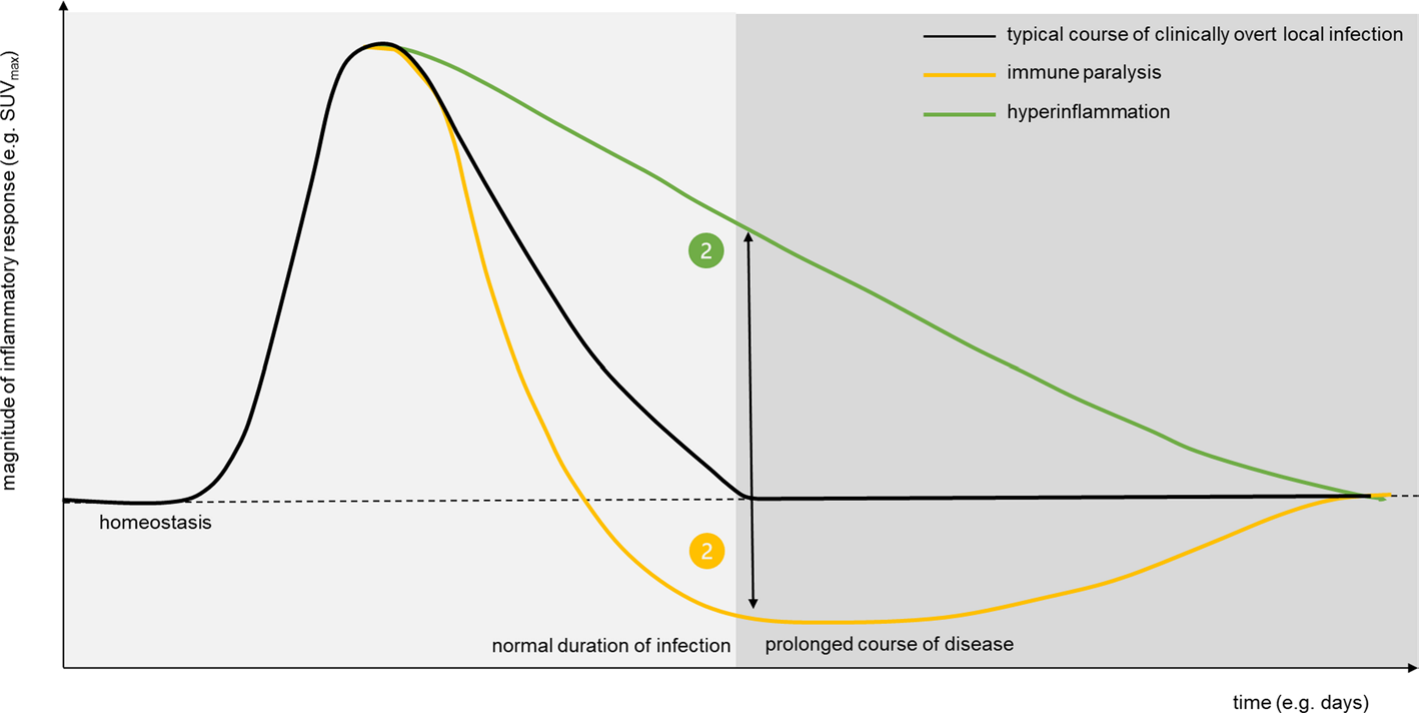
Artificial intelligence to predict outcomes

Immune responses are a complex series of events that involve different immune cell populations and requires a concerted action in multiple body compartments (Spitzer et al. 2017; Chavakis et al. 2019). For example, upon infection, inflammatory monocytes are recruited from the bone marrow and spleen and increased myelo- and granulopoiesis should compensate for the loss of these effector cells in peripheral tissues (Hotchkiss et al. 2016). Indeed, most patients referred for nuclear imaging under suspicion of an infectious or inflammatory condition have symptoms and clinical signs indicative of systemic immune responses, such as fever, increased C-reactive protein and erythrocyte sedimentation rate and leukocytosis. The questions here are whether we can capture these systemic responses using molecular imaging and use this information to improve outcome prediction (Fig. 3).

The switch from a quiescent to an activated status inevitably comes with metabolic reprogramming of immune cells, resulting in increased glycolytic capacity (Netea et al. 2020a; Arts et al. 2017, 2018). As [^18^F]FDG-PET is a highly sensitive technique to quantify glycolysis on a whole-body scale, we and others have demonstrated that increased uptake of [^18^F]FDG-PET in organs involved in hematopoiesis and immune activation, e.g., bone marrow, spleen and vascular system, associates with the state of immune activation (van der Heijden et al. 2020; Bernelot Moens et al. 2016; Valk et al. 2016; Joseph et al. 2017; Stiekema et al. 2019; Ungar et al. 2020; Kalafati et al. 2020). Paralleling mechanisms might also play a role in the responsiveness of immune cells in anticancer immunity (Kalafati et al. 2020; Netea and Joosten 2018; Schwenck et al. 2020; Seith et al. 2020). Increased [^18^F]FDG uptake in bone marrow or spleen, as substrate of systemic immune activation, are associated with improved clinical outcome in melanoma patients under immune checkpoint inhibitors (Seban et al. 2021). This effect could potentially also be observed in overacting autoimmune events by [^18^F]FDG PET (Spitzer et al. 2017; Kalafati et al. 2020; Flint et al. 2017).

In patients with atherosclerotic cardiovascular diseases there is already evidence that increased [^18^F]FDG uptake in the arterial wall, spleen and bone marrow predicts future occurrence of cardiovascular events (Emami et al. 2015). To the contrary, clinical studies in sepsis showed that patients with *decreased* glycolytic capacity in leukocytes have a worse clinical outcome (Cheng et al. 2016; Hotchkiss et al. 2013; Kaufmann et al. 2018), a phenomenon called ‘immune paralysis.’

Thus, as the whole-body field of view of PET allows to assess body compartments involved in the systemic response to infection or inflammation, which element hampers the analysis of these potentially predictive data? At present, the integration of this additional data on immune metabolism in multiple body compartments depends on the limited human capacity to deal with multi-dimensional data. Systems biology studies are integrating large-scale (‘omics’) data, e.g., from different tissues (Kidd et al. 2014) and therefore are urged to implement AI-based technologies for data analysis (Camacho et al. 2018). These studies enabled a more comprehensive mechanistic insight in multidimensional complex diseases (Yang 2020), such as cardiovascular disease (Lempiainen et al. 2018; Makinen et al. 2014; Shu et al. 2017; Slart et al. 2021). These studies demonstrated that the net outcomes on patient level result from perturbations in multiple body compartment involving diverse cell types and molecular pathways. The integration of these different scales of data, in which the contribution of the individual components can vary from subject to subject, demonstrates that cardiovascular disease is promoted by increased inflammatory pathways in the liver, adipose tissue and vascular system (Libby et al. 2019), as well as by the immune response, which is not limited to the arterial wall as it is also detectable in the bone marrow and the spleen.

Similar to infections, effective anticancer immune responses require an integrated action from both innate and adaptive immune cells (Chiossone et al. 2018) including their activation in local and distant body compartments (Spitzer et al. 2017; Kalafati et al. 2020). These observations underscore the general concept that on a systems level, metabolism and immune responses are connected (Flint et al. 2017).

Thus, immune metabolism is a preeminent example of reciprocal interactions on a cellular, organ and system level (Lercher et al. 2020) that impact inflammatory and infectious diseases as well as the homeostasis of the immune system, as will be discussed later.

**Fig. 4:**
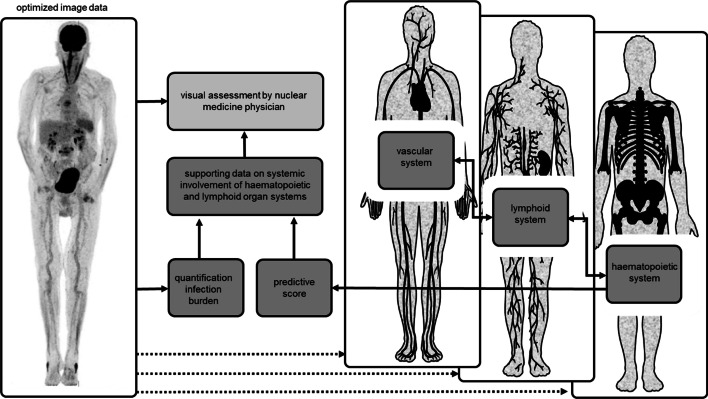
AI could be used to develop a predictive score calculated from the extracted information on the vascular, lymphoid and hematopoietic system. This score characterizes the level of systemic inflammation, for example, in a patient with suspected vasculitis and therefore supports the assessment of the nuclear medicine physician

[^18^F]FDG PET is well-suited to measure metabolic activity across these multiple circuitries, provided that AI-based technologies are developed to extract and process these data in predictive models (Fig. 4).

### Artificial intelligence to provide prognostic information

Pathogen and damage-associated molecular patterns are sensed by cells of the innate immune system, inducing rapid activation and non-specific responses to eliminate the trigger. A growing body of evidence suggests that, in addition to these rapid ‘first line-of-defense’ responses, long-term functional reprogramming of innate immune cells occurs and co-determines responses to subsequent triggers. So, ‘immunological memory’ is no longer considered to be exclusive for cells of the adaptive immune system, but also occurs in innate immune cells, both in hematopoietic progenitor cells (central trained immunity) and in differentiated cells such as monocytes, macrophages and natural killer cells (peripheral trained immunity) (Netea et al. 2020a; Schultze et al. 2021). These ‘trained immunity’ phenotypes have implications for the response to future infections (Netea et al. 2020b). Central in the development of trained immunity is epigenetic reprogramming, which is closely intertwined with metabolic reprogramming, characterized by an increased glycolysis, glutaminolysis and mevalonate pathway, among others. This mechanism allows altered immune-metabolic circuits in immune cells to respond with faster and higher upregulation of aerobic glycolysis and subsequent cytokine production capacity upon subsequent infectious triggers (Dominguez-Andres and Netea 2019).

**Fig. 5:**
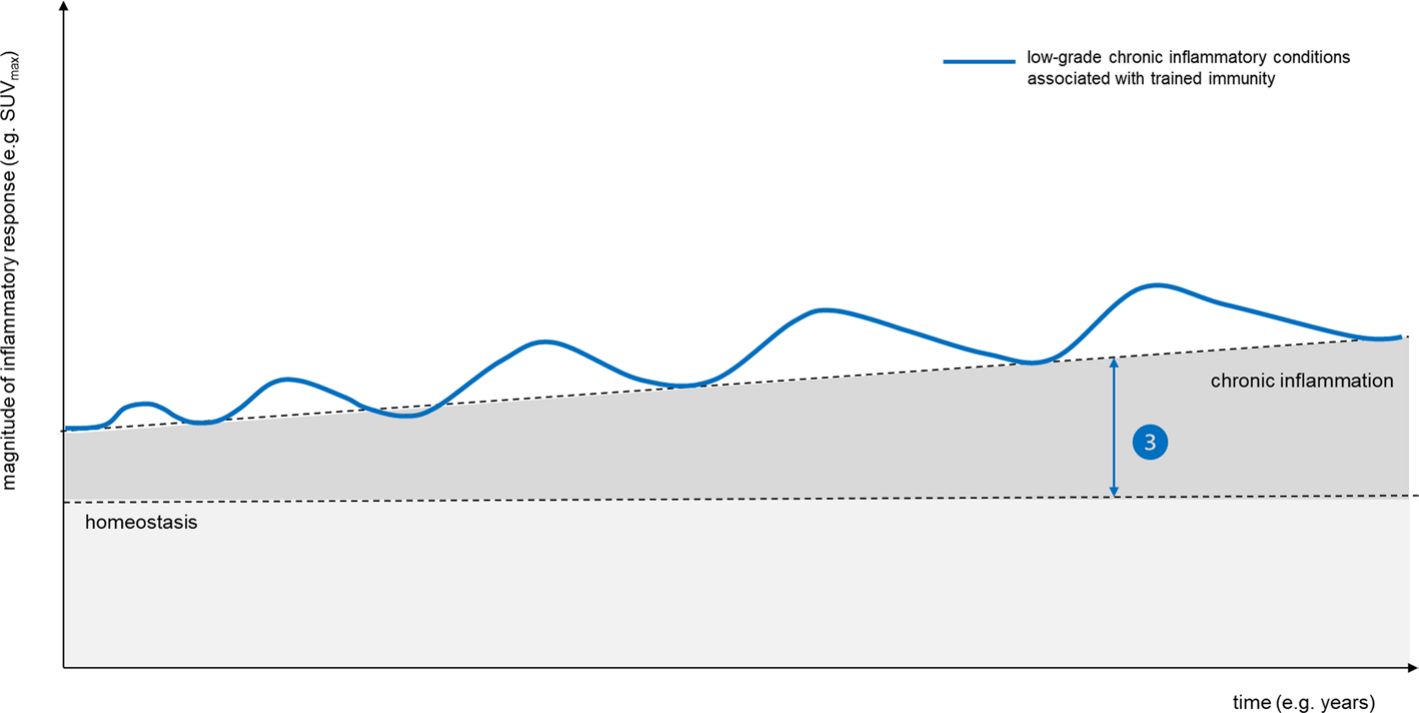
Artificial intelligence to provide prognostic information

Environmental inflictions, and the associated inflammatory response on tissue level, culminate during life span and are considered an integral part of ageing (Lopez-Otin et al. 2013; Sugimoto et al. 2019). It is tempting to speculate that beyond the development of whole body [^18^F]FDG PET as a predictive imaging classifier based on immune metabolic phenotypes, there might be a prognostic role for [^18^F]FDG PET to determine long-term outcome associated with chronic inflammatory conditions (Fig. 5).

**Fig. 6:**
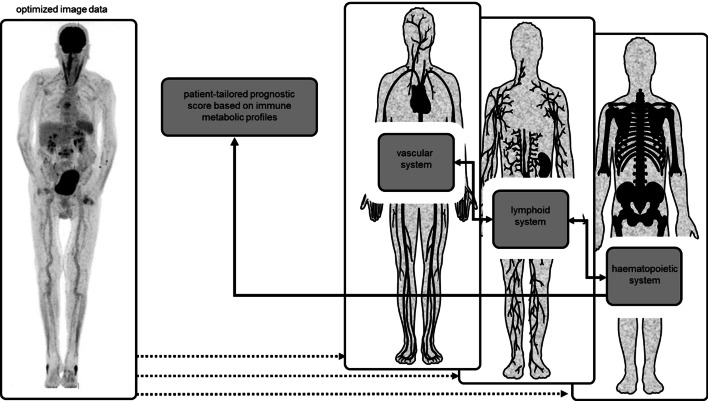
The extracted information about the vascular, lymphoid and hematopoietic system can be facilitated by AI to develop a patient-tailored prognostic score

In addition to trained immune cells, repetitively triggered stromal cells can also convert into a state of chronic low-grade inflammation (Bekkering et al. 2016a, b, 2018, 2019; Leentjens et al. 2018), with detrimental impact on long-term clinical outcomes. For example, endothelial cells of the vascular system, which are key in directing the trafficking of immune cells to inflamed tissues, also respond to systemic inflammatory mediators (Pober and Sessa 2007). Moreover, these endothelial cells are exposed to a multitude of noxes throughout a lifespan, e.g., hypercholesterolemia or hyperglycemia, inducing cell damage and low-grade inflammation aimed to maintain endothelial integrity. The role of [^18^F]FDG PET imaging in large vessel vasculitis is established (Jamar et al. 2013; Slart et al. 2018b) and is currently explored for chronic inflammatory conditions (van der Heijden et al. 2020; Noz et al. 2020; Valk et al. 2017). Defining a threshold on this sliding scale from overt vascular wall inflammation, e.g., in the context of vasculitis, associated with symptoms and representing a clinical entity, to low-grade inflammation, associated with chronic inflammatory conditions such as atherosclerosis, perhaps is a new prognostic task for [^18^F]FDG PET that comes within reach with the advent of AI-based technologies (Fig. 6). Here the gain in image quality of new digital PET scanners and especially of the recently introduced long-axis field of view PET scanner might favor the evaluation of vascular wall inflammation by PET, as it allows to acquire dynamic data that can provide more accurate quantification of the biological process, in this case [^18^F]FDG uptake rates in cell types involved in vascular wall inflammation. Secondly, it can assess the involvement of primary and secondary lymphoid organs throughout the whole body in systemic diseases versus tissue-confined local inflammatory process.

Similarly, as defects in metabolism are commonly associated with impaired outcomes in various conditions, such as impaired regulation of glucose homeostasis in type 2 diabetes (Bernelot Moens et al. 2016; Lee et al. 2018; Hotamisligil 2017; Norata et al. 2015) or obesity in anticancer immunity (Thaiss et al. 2021; Ringel et al. 2020) and atherosclerosis (Bucerius et al. 2012, 2014), such prognostic role might be requested from [^18^F]FDG by clinical disciplines in the near future. As far as it concerns the immune system, this field is actively researched to develop therapeutic strategies (Mulder et al. 2019) to enhance or reduce inflammatory responses in anticancer immunity (Priem et al. 2020), autoimmune (Municio and Criado 2020) and infectious diseases (O'Neill and Netea 2020; Netea et al. 2020c). The higher sensitivity of the whole-body PET scanner enables the acquisition of low-dose PET images below 1–1.5 mSv, allowing its more frequent use in non-oncologic diseases for risk stratification assessment.

How should AI-based technologies be implemented to facilitate the development of [^18^F]FDG PET as a tool to determine prognostic immune metabolic profiles? The challenge in such task lies in the discrimination of *bona fide* inflammation from *mala fide* inflammation. This difference is expected to be subtle, as the inflammatory response in a distinct context of disease is beneficial rather than pathological, and temporally apart from an identified trigger.

Accurate appraisal of subtle differences requires large datasets for training and supporting ‘circumstantial evidence’ where possible. For example, it can be postulated that assessment of [^18^F]FDG uptake in the vascular wall as *mala fide* will be more accurate if not only metabolic activity in the hematopoietic system is taken into account, but also atherosclerotic calcifications and body composition in terms of subcutaneous and visceral adipose tissue versus muscle mass can be deduced from the low-dose CT (Laur et al. 2021) and incorporated in the risk assessment. Similarly, AI-algorithms are available to assess bone mineralization and emphysema score on low-dose CT (Ebrahimian et al. 2021), which could determine the host’ long-term responses to environmental inflictions like smoking. As for now, such information is not incorporated in current practice yet. Along with training AI-algorithms on large datasets come the need for harmonization, smart processing and modelling, each individual task is suited for AI-based technologies. In line with developments in CT imaging (Choe et al. 2019), AI-based algorithms can be trained to overcome center- or vendor-related differences in reconstruction settings (Arabi and Zaidi 2021) and allowing to extract radiomic features (Orlhac et al. 2018; Zwanenburg 2019).

Reiterating from the conceived potential of AI to transform healthcare (Denny and Collins 2021), contemplating whole body [^18^F]FDG PET images as huge interoperable datasets that meet the criteria of diversity and inclusion, implies that we need AI-technology to open up these big datasets and exploit its potential to approach immune metabolism on a systems level in clinical settings.

## Challenges and potential solutions

Despite the sheer limitless methodological and technological advancements in AI-based technology, the widespread application of AI-tools in molecular imaging of infection and inflammation is facing some major challenges on its way into routine clinical use. One hurdle that will need to be overcome is to deal with the ‘black box’ stigma on AI-based algorithms; the lack of explainable correlations between in- and output leaves physicians often hesitant to rely on AI-based output. In addition, the subtle differences between physiological and pathological and potentially high variations between individual patients requires large datasets and/or labeled datasets based on ground truth, of which the latter is more difficult as it will require invasive procedures to obtain tissue samples to analyze immune cells’ metabolic profiles.

To address these issues, smaller studies with high translational design including flow cytometry, metabolomics and/or transcriptomic data from circulating immune cells or the hematopoietic system could provide proof-of-concept to correlate specific imaging findings to immune metabolic features in relevant cell populations (e.g., Hotchkiss et al. 2016). Subsequent studies can then build further upon these data and provide larger datasets for validation and to determine its value in real-life clinical setting. For such studies with large datasets, questions on harmonization of input data arise, which have partly been tackled in the EARL program for multicenter studies by the European Association for Nuclear Medicine. Moreover, computing higher order features from PET requires image normalization during data processing and training AI-based models on a wide range of scanner hardware can provide a solution that would be compatible with current deep-learning networks, provided that the computing power is sufficient. Nevertheless, the input for predictive or prognostic AI-based models as discussed in this communication should be ‘supervised’ as only PET parameters computed from predefined immune relevant organ systems, in line with current concepts on immune-metabolism, should serve as input data.

Another challenge will be the integration of the clinical experience from the nuclear medicine and radiology readers into a future AI-supported workflow of clinical decision making. The experience of the reader, who is also taking the case-specific clinical context into account, will be difficult to replace. Therefore at least in the coming few years, AI might support the clinical decisions if it is confirming the evaluation by the reading physician, but it is unclear how to proceed if human and AI-based assessment are coming to the contradictory results. In line with broader developments of AI-based technology in medical imaging, liability issues need to be addressed in the near future.

## Conclusion

AI tools are increasingly used for a growing number of tasks in the imaging field ranging from technical applications which improve the sensitivity of scanners to biomedical applications for holistic data analysis. As proposed above, AI has the potential to improve the detection of inflammatory diseases and predict prognosis and outcome of patients under various immune-mediated conditions (Table 1). Furthermore, these tools are capable to provide a deeper understanding of the basic molecular mechanisms of inflammatory diseases.

**Table 1:** Key elements for the future: opportunities, challenges and solutions of AI in infection and inflammation molecular imaging

	Opportunities	Challenges	Solutions
Improve detection	Detection of low-grade or localized infections	Insufficient spatial resolution Lack of sensitivity Long acquisition times	Cardiac and respiratory motion correction Improving of the detector sensitivity. e.g., by predicting the depth-of-interaction of incoming photons Image denoising by AI
Predict outcomes	Prediction of individual outcome by assessing the systemic immune response	Validated data derived from multiple organ systems required	In depth analysis of high-dimensional imaging data by AI algorithms Large-scale prospective trials including in vitro ‘omics’ data
Provide prognostic information	Imaging as predictive classifier to determine long-term outcome	Discrimination of physiological vs. pathological immune metabolic pathways Subtle differences require large datasets for training High efforts for data harmonization	AI analysis on big data provided by, e.g., large multicenter studies or national health care providers

For a successful application in future health care in the context of personalized medicine the tight integration of the AI imaging tools with other diagnostic methods like genetic analysis, proteomics and metabolomics is the key to achieve reliable and impactful data which improves treatment decisions and ultimately patients’ well-being and survival.

## Data Availability

None.

## Abbreviations

AI: Artificial intelligence
CT: Computed tomography
[^18^F]FDG: 2-[^18^F]fluoro-2-deoxy-D-glucose
LOR: Line-of-response
MRI: Magnetic resonance imaging
PET: Positron emission tomography
SPECT: Single-photon emission tomography
SUV: Standardized uptake value
